# Impact of extreme weight loss on factor VIII concentrate pharmacokinetics in haemophilia

**DOI:** 10.1136/bcr-2020-238036

**Published:** 2021-04-12

**Authors:** Iris van Moort, Laura H Bukkems, Laurens Nieuwenhuizen, Marjon H Cnossen

**Affiliations:** 1 Department of Paediatric Haematology, Erasmus University Medical Center—Sophia Childrens Hospital Rotterdam, Rotterdam, The Netherlands; 2 Department of Clinical Pharmacology—Hospital Pharmacy, Amsterdam UMC Locatie AMC, Amsterdam, North Holland, The Netherlands; 3 Department of Hematology, Maxima Medical Centre Location Veldhoven, Veldhoven, Noord-Brabant, The Netherlands

**Keywords:** haematology (drugs and medicines), haematology (incl blood transfusion), pharmacokinetics

## Abstract

We explored the effects of extreme weight loss after gastric bypass surgery on factor VIII concentrate pharmacokinetic (PK) parameters in a patient with haemophilia A. We present a 32-year-old man with severe haemophilia A, with a body mass index (BMI) of 42.6 kg/m^2^ who underwent laparoscopic sleeve gastrectomy. We showed that a population PK model with ideal body weight as morphometric variable instead of bodyweight led to an adequate description of the individual PKs in this patient with a variable BMI. Strikingly, no differences were observed in the individual PK parameters after extreme weight loss. Therefore, the resulting extreme weight loss after surgery did not lead to prophylactic dose changes in this patient with severe haemophilia. We carefully conclude that population PK–pharmacodynamic models are still obligatory to give more insight into functional effects of significant weight loss on the haemostatic balance.

## Background

Haemophilia A is an X-linked inherited bleeding disorder caused by a deficiency in coagulation factor VIII (FVIII). To prevent spontaneous bleeding in muscles and joints, patients with severe and some moderate haemophilia A receive FVIII prophylactic replacement therapy. In clinical practice, FVIII concentrate dosing is still mainly based on bodyweight.^1^ As overweight and obesity are a growing global health problem with a current prevalence of 43.3% in the adult European and North American haemophilia population, appropriate dosing strategies for replacement therapy in this patient group are relevant to safeguard treatment costs without loss of quality of care.^2^

In several studies by Henrard *et al*, in vivo recovery (IVR) has been shown to be significantly higher in overweight and obese patients with haemophilia than in normal weight patients.^3–5^ In addition, weight-adjusted clearance decreases with age, whereas weight-adjusted volume of distribution does not.^6^ The latter suggesting that weight-adjusted volume of distribution is constant over time.^3 4 6^ We set out to further prove this assumption by describing the impact of extreme weight loss on FVIII pharmacokinetic (PK) parameters in haemophilia A, which has not been done earlier. Recently, a patient with severe haemophilia A was reported who safely underwent a laparoscopic mini gastric bypass operation for weight reduction without details on FVIII PK parameters.^7^ We are the first to describe such a surgical intervention in a patient with haemophilia A, including analyses of FVIII PK parameters.

## Case presentation

We present a 32-year-old man with severe haemophilia A (FVIII<0.01 IU/mL), with a bodyweight of 133.5 kg, and body mass index (BMI) of 42.6 kg/m^2^. Patient was planned for laparoscopic sleeve gastrectomy after extensive clinical, laboratory and psychological testing and individual FVIII concentrate PK profiling. Consequently, a PK-guided perioperative loading dose and subsequent dosing regimen were calculated. Six months later surgery was performed. At the day of surgery, patient’s bodyweight was 142.0 kg with a BMI of 45.3 kg/m^2^. FVIII levels were monitored daily perioperatively and dosing was iteratively adjusted by application of *maximum a posteriori* (MAP) Bayesian analysis.^8^ Surgery was performed without complications, more specifically without (peri)surgical bleeding. He was discharged from the hospital after 4 days and received additional FVIII doses until postoperative day 10, at which moment patient resumed FVIII prophylaxis.

## Investigations

Preoperatively, a PK profile was obtained after infusion of 5000 IU (37.4 IU/kg) of recombinant FVIII (NovoEight) (t=0). FVIII measurements were performed by one-stage assay at respectively t=4 hours, t=48 hours and t=52 hours after infusion.^9^ The Sysmex CS 5100 (Sysmex, Kobe, Japan) was used for the one-stage assay combined with following reagents all from Siemens (Siemens Healthcare Diagnostics, Marburg, Germany): FVIII Actin FS, FVIII deficient plasma and Standard Human Plasma as a calibrator. A FVIII concentrate washout period or correction for the preadministration FVIII levels was not necessary, as both timing and dose of three previous FVIII concentrate infusions were recorded. Individual PK parameters were calculated by MAP Bayesian analysis in NONMEM V.7.4.1 (ICON Development Solutions, Ellicott City, Maryland, USA) using our prophylactic population PK model including overweight and obese patients, with ideal body weight (IBW) as morphometric variable.^10 11^ PK profiling was repeated 6 and 12 months after surgery to investigate impact of weight loss on patient’s FVIII PK parameters.

## Outcome and follow-up

### FVIII concentrate PK parameters

**Figure 1:**
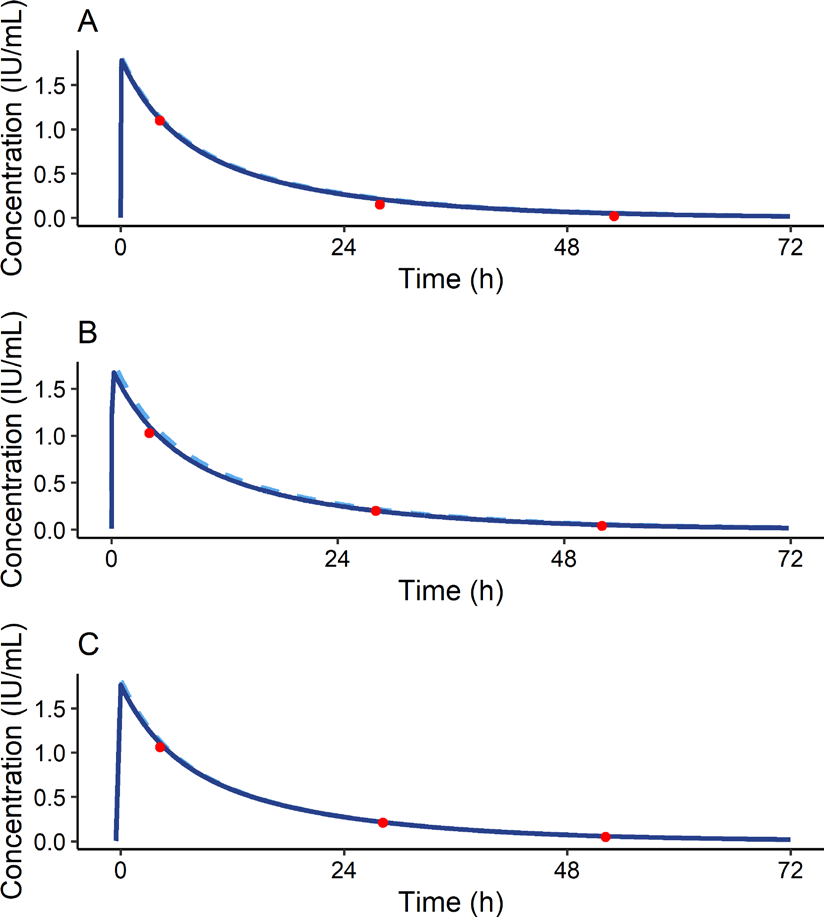
Individual pharmacokinetic (PK) profiles before, 6 months after and 12 months after bariatric surgery. The dark blue line depicts individually predicted factor VIII (FVIII) activity levels after a dose of 5000 IU FVIII concentrate. The interrupted light blue line depicts the population values. The red dots are the measured FVIII levels. (A) PK profile before surgery with a body weight of 133.5 kg (BMI 45.3 kg/m^2^). (B) PK profile 6 months after the patient’s surgery with a body weight of 110.4 kg (BMI 35.3 kg/m^2^). (C) PK profile 12 months after the patient’s surgery with a body weight of 106.4 (BMI 34.0 kg/m^2^).

**Figure 2:**
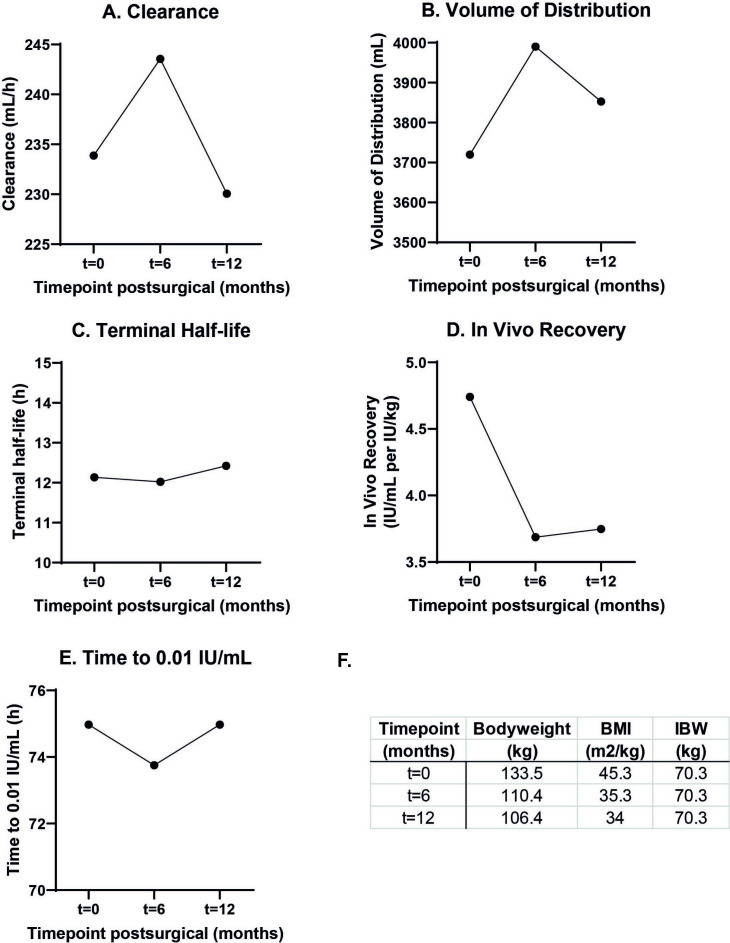
Individual pharmacokinetic parameters before surgery (t=0), 6 months after surgery (t=6 months) and 12 months after surgery (t=12 months). (A) Clearance, (B) volume of distribution, (C) terminal half-life, (D) in vivo recovery, (E) time to 0.01 IU/mL, (F) table summarising the morphometric variables measured at each time point.

Six months after surgery, bodyweight decreased with 31.6 kg, from 142.0 to 110.4 kg, with BMI decreasing to 35.3 kg/m^2^. A PK profile was repeated to assess individual PK parameters. One year after surgery when final PK profiling was performed, patient weighed 106.4 kg with a BMI of 34.0 kg/m^2^. Figure 1 shows individual PK curves at each time point with IBW (70.3 kg) as a morphometric variable. As depicted, measured FVIII levels follow predicted FVIII levels, confirming a good fit of the model to the data by MAP Bayesian analysis. The influence of weight loss on individual PK parameters is visualised in figure 2. Figure 2D shows that IVR decreased significantly with decreasing bodyweight. Strikingly, FVIII clearance and volume of distribution remained similar over time (figure 2A, B), resulting in a similar half-life over time (figure 2C).

### FVIII concentrate dosing and trough level simulations

As a prophylactic dose of 25–40 IU/kg is recommended by the World Federation of Hemophilia,^1^ time to trough of 0.01 IU/mL was calculated after a hypothetical prophylactic FVIII dose of 3500 IU (26 IU/kg before bariatric surgery). Simulations using the patient’s individual PK parameters showed that time to 0.01 IU/mL was not subject to change (figure 2E). After weight loss, a novel optimal prophylactic dosing schedule was calculated and the original prophylactic regimen of 750 IU (now 7 IU/kg) every other day remained adequate.

## Discussion

The present case was analysed to determine impact of extreme weight loss on FVIII PK parameters. PK profiling before and after gastric bypass in a patient with severe haemophilia A strikingly did not differ with regard to calculated individual PK parameters and therefore did not lead to dose changes of prophylaxis.

Although extreme weight loss did not lead to alterations of individual FVIII PK parameters, it is important to realise that weight reduction may lead to shifts in haemostatic balance leading to clinically relevant presentations. Several reports have described changes in both procoagulant and anticoagulant factors. Overall, obese individuals are thought to be prothrombotic due to lower fibrinolytic potential caused by higher plasminogen inhibitor levels, leading to decreased clot lysis and overall bleeding tendency may be lower.^12 13^ Hypothetically after extreme weight loss, patients may experience more bleeding due to normalisation of fibrinolysis, subsequently needing higher prophylactic FVIII concentrate doses due to increased bleeding. Contrastingly, it has also been reported that 1 year after gastric bypass surgery, antithrombotic protein levels are also lower.^14^ Future PK–pharmacodynamic (PD) studies should evaluate influence of obesity and weight loss on haemostatic balance to establish its relevance.

In previous studies, it has been suggested that IBW, as calculated according to Lorentz’s formula including height and sex and not total body weight, should be applied to minimalise interindividual differences in FVIII PK and to concomitantly reduce factor concentrate consumption and decrease treatment costs.^10 11 15^ In addition, lower amounts of factor concentrate could be beneficial if administrated in lower and middle income countries. Moreover, as the extended half-life products are increasingly available for haemophilia A, using IBW may also be cost attractive in case of these newer, often more expensive, products. In this case report, we additionally propose that IBW may be of value to compensate for intraindividual differences in FVIII PK when bodyweight is variable. Figure 1 shows the three individual PK profiles at consecutive time points with varying bodyweight, fitted with IBW as a morphometric variable to describe alterations in FVIII PK after weight loss. IBW estimates volume of distribution optimal, both before and after weight reduction and estimates FVIII peak levels accordingly. This can be explained physiologically as FVIII concentrate is infused into the vascular space. This is supported by the fact that volumes of distribution approximate plasma volume. Therefore, weight loss does not affect volume of distribution. Furthermore, FVIII clearance did not change over time, which we have also demonstrated in prior reports on interindividual variation in FVIII PK.^11^ This case report shows that a population model with IBW as morphometric variable allows an adequate description of the individual PKs in a patient with varying BMI.

In conclusion, obesity is a growing, global healthcare problem, also affecting patients with haemophilia. Extreme weight loss does not result in altered individual PK parameters and there does not seem to necessitate adjustment of perioperative and prophylactic dosing regimens based on PK. However, monitoring of bleeding and ultimate construction of population PK–PD models are still obligatory to define effects of weight loss on haemostasis.

**Patient’s perspective**

I was of course born with hemophilia A. Due to my overweight, which has further increased the last few years, I recently made the difficult decision with the hemophilia treatment team to have bariatric surgery performed. The main reason for this decision were my concerns regarding my general health. I shared my anxiety for the operation due to my bleeding disorder with my doctor and the nurses. They were very supportive, and assured me that they would collaborate closely with the surgeon to organize the necessary replacement therapy to prevent any perioperative bleeding.

A few months before surgery, I was able to participate in a clinical research project for example, the randomized controlled perioperative OPTI-CLOT trial in hemophilia A patients undergoing surgery. In this trial, standard dosing based on bodyweight is compared to an innovative strategy to individualize factor VIII concentrate dosing by looking at the velocity with which the factor concentrate disappears from the circulation, also called pharmacokinetic (PK)-guided dosing. This approach intrigued me and I decided that I very much wanted to participate. Later, when the research coordinator asked me to participate in a this small substudy, I gladly agreed. Understanding that bariatric surgery in a severe hemophilia A patient is rare and educative. The research team wanted to investigate the effect of extreme weight loss on the PK of the administered factor VIII concentrate. I experienced this as a unique chance to personalize my own treatment and to optimize therapy for other patients with a severe bleeding disorder. Especially as the number of overweight hemophilia patients is steadily increasing as I understood from the research coordinator. During the operation and after surgery, my factor VIII levels would be monitored extensively, which also made me feel safe that factor VIII levels would be sufficient to prevent bleeding.

One year after my bariatric surgery, I discussed the results of the study with the research coordinator. It was concluded that that the extreme weight loss I experienced was not of significant influence on the PK of the administered factor VIII concentrate. To be honest, I expected the contrary and I understood that the research team was also surprised by these results. I realize that this is exactly what the importance is of such studies as hypotheses can be tested and are sometimes shown to be untrue. I am glad to have been able to contribute to the ultimate aim of the study group to personalize treatment in patients with a bleeding disorders. I would be happy to participate in future research projects as I have found the whole escapade very special. I would like to very much thank the hemophilia and surgical team for all their care and organization!

**Learning points**

- The prevalence of overweight and obesity is increasing rapidly in patients with haemophilia A.
- Extreme weight loss does not influence factor VIII concentrate pharmacokinetics in haemophilia A.
- As the haemostatic balance changes after extreme weight loss, monitoring of pharmacodynamics becomes more relevant than pharmacokinetics.
